# A Novel Morphometry-Based Protocol of Automated Video-Image Analysis for Species Recognition and Activity Rhythms Monitoring in Deep-Sea Fauna

**DOI:** 10.3390/s91108438

**Published:** 2009-10-26

**Authors:** Jacopo Aguzzi, Corrado Costa, Yoshihiro Fujiwara, Ryoichi Iwase, Eva Ramirez-Llorda, Paolo Menesatti

**Affiliations:** 1 Institut de Ciències del Mar (ICM-CSIC), Passeig Marítim de la Barceloneta 37-49, 08003 Barcelona, Spain; E-Mail: ezr@cmima.csic.es (E.R.-L.); 2 AgritechLab - Agricultural Engineering Research Unit of the Agriculture Research Council, Via della Pascolare (CRA-ING), 16, Monterotondo (Rome) Italy; E-Mail: paolo.menesatti@entecra.it (P.M.); 3 Japan Agency for Marine-Earth Science and Technology (JAMSTEC), 2-15 Natsushima-Cho, Yokosuka, Kanagawa 237-0061 Japan; E-Mails: fujiwara@jamstec.go.jp (Y.F.); iwaser@jamstec.go.jp (R.I.)

**Keywords:** automated video-image analysis, deep-sea, behavioural rhythms, mudflows, inertial currents, internal tides, cold-seeps, Sagami bay

## Abstract

The understanding of ecosystem dynamics in deep-sea areas is to date limited by technical constraints on sampling repetition. We have elaborated a morphometry-based protocol for automated video-image analysis where animal movement tracking (by frame subtraction) is accompanied by species identification from animals' outlines by Fourier Descriptors and Standard K-Nearest Neighbours methods. One-week footage from a permanent video-station located at 1,100 m depth in Sagami Bay (Central Japan) was analysed. Out of 150,000 frames (1 per 4 s), a subset of 10.000 was analyzed by a trained operator to increase the efficiency of the automated procedure. Error estimation of the automated and trained operator procedure was computed as a measure of protocol performance. Three displacing species were identified as the most recurrent: Zoarcid fishes (eelpouts), red crabs (*Paralomis multispina*), and snails (*Buccinum soyomaruae*). Species identification with KNN thresholding produced better results in automated motion detection. Results were discussed assuming that the technological bottleneck is to date deeply conditioning the exploration of the deep-sea.

## 1. Introduction

The identification of species and their behavioural rhythms in deep-water continental margins and deep-sea areas is of actual and elevated importance for fishery management and biodiversity estimation [1]. To date, the most widely employed system for the study of communities at these depths is bottom trawl surveying (reviewed in [2]). Anyway, species rhythmic movements in relation to recognised geophysical cycles such as the fluctuations in light intensity (day-night based) or in hydrodynamism (internal-tide based) may bring animals in and out from punctual trawl sampling windows [3]. This phenomenon could consistently alter the estimation of species biomasses and areas of distribution of their populations [4]. While the effects of these geophysical cycles on costal species is to date widely studied and hence recognized, this environmental rhythmic regulation is mostly unknown in slope, rises and abyssal plain areas [5].

The understating of ecosystem dynamics in deep-water continental margin areas and the deep-sea is to date still limited by technical and economic constraints that affect sampling repetition [6]. A solution is represented by automated collection of data by a wide array of sensors located in permanent submarine stations. In the past two decades, the number of these stations bearing video cameras has progressively increased along with the socio-economic interest for the exploration of the sea [7]. The use of video filming techniques for species recognition and behavioural study was then attempted in coastal areas (reviewed in [8]). Anyway, the use of this technique for the study of behavioural rhythms remains to date largely underexploited at any depth, especially in the deep-sea. The reasons for this are mainly related to the lack of automation in footage analysis. This is chiefly due to added contextual difficulties such as the absence of environmental light and the presence of variable fouling [9]. This obliges researchers to the manual examination of thousands of images [10,11].

In this context, the development of adequate analytic protocols based on automated video-image analysis may represent an important and challenging step toward the study of species presence (by recognition), as well as their rhythmic behaviour in the deep-sea. This is particularly important for benthic (i.e., demersal) communities in poorly accessible environments of elevated ecological and geological interest such as those of cold-seeps and hydrothermal vents [12]. Accordingly with this scenario, we elaborated a novel morphometry-based protocol of automated video-image analysis to identify the benthic fauna and their associated behavioural rhythms in the cold-seep clam field of Sagami Bay (1,100 m depth). That protocol was customized to track animal movement by frame subtraction and to perform species recognition by Fourier Descriptors analysis on different animals' profiles. We analysed the one-week footage taken by a video camera of the local permanent observatory. Outputs of video-image analysis as time series of visual counts were treated with the protocols of biomedical chronobiology in order to validate the feasibility of the method for the study of behavioural rhythms.

## 2. Materials and Methods

### 2.1. The Footage

One-week footage lasting from 09-04-99 to 16-04-99 was taken by a submarine infrared 3CCD video-camera mounted on the “*Real-Time Deep-Sea Floor Permanent Observatory*” [13]. That permanent station is located in a cold seep clam field (i.e., *Calyptogena soyoae*) at 1,100 m depth off Hatsushima Island, in Sagami Bay (34°59.97′N, 139°13.69′E; central Japan). The video-camera was continuously acquiring images in a time-lapse mode (i.e., a frame each 4 s) under a constant source of illumination (i.e., six white-light lamps). Videos were stored on VH-S videotapes and their processing in relation to behavioural rhythms was never attempted before. We selected long lasting continuous 10 years-old footage for the technological challenge of performing automated video-image analysis on videos lacking digital standards. The footage was digitized and partitioned into frames at a rate equivalent to the frequency of video recording. Footage processing and video-image analysis were both carried out with MatLab 7.1.

### 2.2. Video-image Analysis

Motion detection procedure identified animals based on their displacement through consecutive frames [14-17]. In this process, the quality of extracted information depends upon several contingent factors typical of the deep-sea context [9]. Firstly, the detection of movement depends upon the rate of image acquisition in comparison to the speed of animals' motion. Secondly, a source of continuous white illumination, gradually decreasing over the distance (i.e., within 3–4 m) is always present during filming operations. This may impair animal detection depending on its positioning within the camera field. Thirdly, consistent water turbidity is often present (i.e., high-contrast organic debris as “marine snow”), creating difficulties in the automated identification of moving animals. Fourthly, different species possess different shapes that are also variables according to animal displacement within the camera filed.

**Figure 1.**
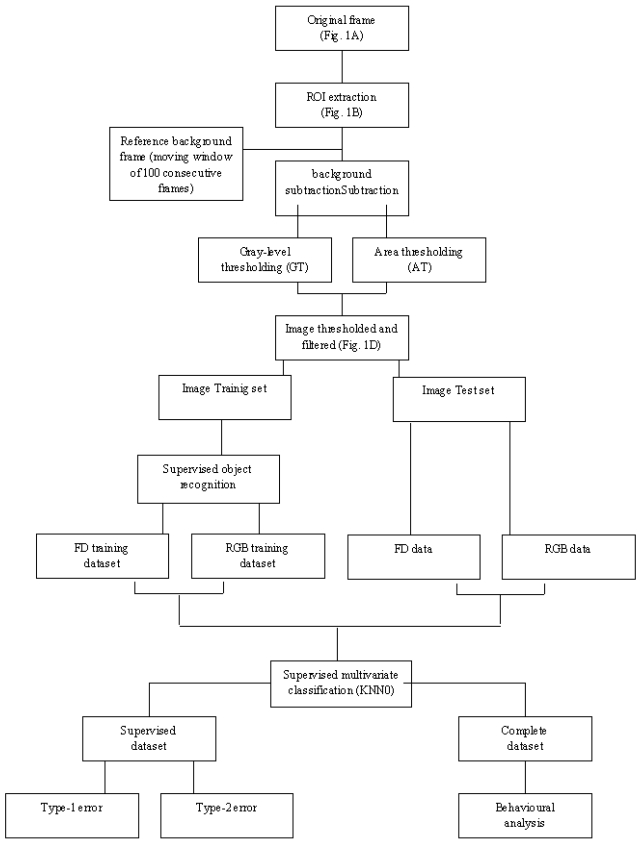
Flow chart representing the different steps involved in the automated procedure.

In spite of all these considerations, an automated video-image analysis protocol was developed according to two major steps: (1) animals' motion detection, by means of image extraction; (2) animals' recognition within different species categories, by multivariate morphometric techniques such as Fourier Descriptors (FD) and the Supervised Standard K-Nearest Neighbours (KNN) analyses. A flow chart specifying the different steps involved in the automated procedure is illustrated in Figure 1.

### 2.3. Animals' Motion Detection

**Figure 2.**
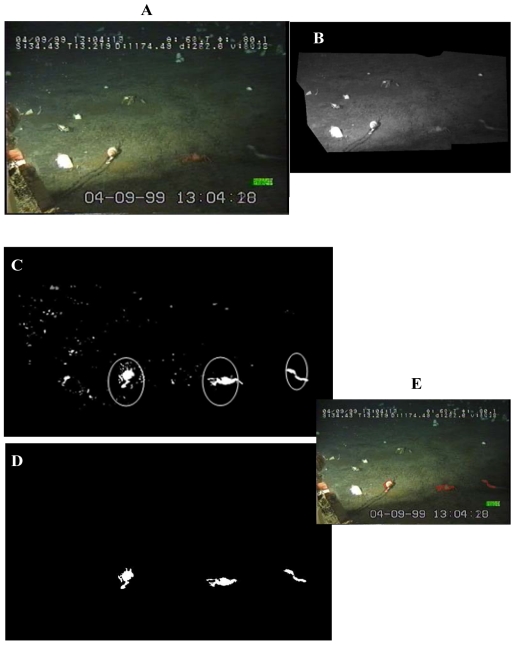
The consecutive stages of automated video-image analysis for the tracking of movement. A frame is firstly subtracted by its consecutive (A). The image without sensible objects is used for background subtraction (B), which occurs within a region of interest (ROI) that delimits the sensible field of view for the motion analysis. The GT and AT thresholding is then performed (see explanations in the text). At this stage, biological objects (i.e., animals) which moved in comparison to the precedent frame (within the circle), are identified (C). Resulting binary image is obtained after GT and AT application (D). The original greyscale image with identified objects in overlay is then saved for later comparisons (E).

Automated video-image analysis for the tracking of movement was based on combined frame subtraction and a multiple filtering procedure (Figure 2).

We applied a simple algorithm in order to subtract the current image for a background image of reference. That reference was constructed by averaging 100 images before it, in order to eliminate the excessive noise [18]. Then, for each frame (Figure 2A) a specific region of interest (ROI) was identified as the central area within the camera field (Figure 2B) in order to exclude all display scripts appearing within the video-corners. Then, the resulting image was filtered for: the area (AT, Area Thresholding), by eliminating objects with sizes outside 150-1,000 pixels range (Figure 2C); the grey-level scale (GT, Grey Thresholding), by applying a single threshold value of 10 (i.e., 8 bit-greyscale level); and finally, the red colour (i.e., according to the Red-Green-Blue, RGB, scale of reference) (Figure 2D). Identified object profiles were then superimposed to the original frame in order to show the correct image extraction (Figure 2E).

### 2.4. Animals' Recognition

FD analysis is a novel protocol used in ecological morphometry when the variation in the outline of different species is studied in relation to differences in their ecology [reviewed by 19]. This analysis is based on the scalability of a curve describing the organism shape: varying the number of used Fourier coefficients, different levels of approximation of the Fourier function to the object shape (as closed contour) can be obtained. FD's were obtained from the Fourier transformation of a complex function representing the object outline in pixel coordinates [20]. The shape of a displacing animal was identified and its perimeter coordinates were calculated in relation to *xy*-ROI field. FD values were normalized and transformed to be size, orientation and translation independent. 20 descriptors were used corresponding to 38 coefficients. Each targeted shape was acquired by FD analysis as implemented by a customized script in MatLab 7.1 (modified from [18]).

**Figure 3.**
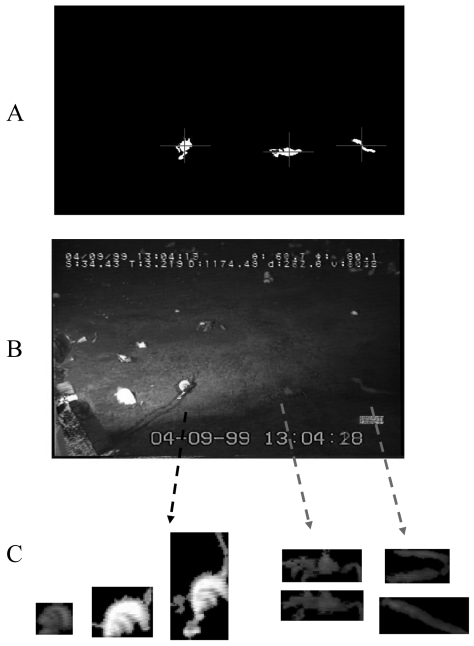
Different steps of species recognition by the attribution of sensible objects (i.e., moving animals) to different classes trough the trained operator supervision: identification of unknown biological objects (A); class attribution (B); and finally, the saving of newly classified objects as single images for their later individual processing by Fourier Descriptors analysis (C). In this example, classified objects are the clam, the red crab, and the eelpout fish of Figure 2.

To increase the efficiency of our automated protocol for species identification and animals' movement tracking, we applied a hybrid supervisionate approach: a continuous and limited subset of frames (i.e., 10,000 images out of 150,000, equivalent to 6.6 % of the total) was randomly selected and analyzed by a trained operator (Figure 3).

Within each frame, object manual selection was carried out trough an interactive script elaborated MatLab 7.1. Moving animals were identified and the software allowed to assigns a membership class (i.e., species) to each of them trough a specific widow-tool. After that manual classification, objects were saved into a reference library as RGB binary images (the average RGB value for each object was used). A training database made by an X-block matrix of FD's and a Y-block matrix made by each corresponding class was created. 164 objects, comprehensively belonging to the three most recurrent species as different categories (see later) were used as training dataset.

KNN is a multivariate clustering method used for classification in morphometry [21-23]. Objects are classified within a membership class in relation to their neighbours by a score computed from Euclidean Distances. The neighbours were taken from the training set (for which the correct classification was proven by the trained operator). For the purposes of the present analysis, a major requirement was not only to classify already identified animals but also to reject unknown objects (outliers). Standard KNN was modified according to our class-modelling purposes to allow classification of unknown objects either into one of the already existing classes or to no classes. A threshold value acting on the Euclidian distance (EDT) was inserted to drive the classification of an object to one or no classes (0). Therefore, we named the modified algorithm “KNN0” (Table 1).

Each new object of the test set, i.e., the whole video excluding the training frames, was classified twice, applying two different KNN0 analyses, one for RGB coordinates and the other for FD coefficients. A reiterative procedure was necessary to establish the more efficient Euclidean Distance Thresholding (EDT): 4.5 for RGB coordinates; 0.2 for FD coefficients. Final single class attribution was obtained by means of a complex logical function depending for the class type, as reported in Table 1. All-TRUE logical conditioning for final class attribution was applied. Any other different condition, determined final class attribution as “null” (the 0 category).

Error estimation on species recognition and sensible object detection (i.e., moving animals) by the automated video-image analysis procedure was calculated in comparison with results provided by the trained operator. Error typologies by KNN were subdivided into: object identification and object classification. The occurrence of two different errors was determined: Type-1 error when an object was not detected; Type-2 error when an object was confused with another one. The latter type of error is the most dangerous since it creates a double error on different species that are then confused each other with a consequent mismatch of their tracked trajectories.

### 2.5. Behavioural Analysis

Time series of visual counts for 4 s for the most recurrent identified species were binned per 10 min interval and a 2-step moving average was conducted in order to reduce high frequency noise. Resulting data sets were then represented in the domain of time by double plotted actograms where consecutive days are placed in a column. The column is then replicated (by repeating each day twice; double plotted) to allow the visual assessment of rhythmicity trough the method of regression line (i.e., eye fitted) onto activity onsets over consecutive cycles (e.g., [15]).

Lomb–Scargle periodogram analysis allows the robust detection of periods in time series of potentially noisy pattern such as those proceeding from field works, when population rhythms are under investigation [24]. That method is a powerful way to find, and test significance of, weak periodic signals in non-evenly spaced time series. We used it (although our time series had no missing values) since it is based on the calculation of the normal Fourier power by spectral decomposition, which is sensible to overall fluctuations in data sets of short duration (i.e., up to a week). We performed this analysis with the “El Temps” software (Prof. Diez-Noguera, University of Barcelona-UB, Spain). The time window of screening was comprised between 600 and 1,500 min (10 h and 25 h) in order to cover a wide range of diel periodicities encompassing frequencies of inertial (atmospheric driven) and tidal (astronomical driven) phenomena (e.g., [3]). In output periodogram plots, the highest significant (p < 0.01) peak represented the maximum percentage of total data variance explained by the inherent dominant periodicity. Periodicity was indicated by that peak value.

The trajectories of displacement of different species were computed to account for ethological differences in the way of movement, as well as their reaction to the video-camera light field. The trajectory of animals that appeared for more than 20 consecutive frames were selected for the analysis of the displacement direction. The borders of illuminated area were graphed onto the ROI *xy*-coordinates plots in order to evidence differences in computed trajectories.

## 3. Results

In this work, the automated processing of the VH-S footage required its fragmentation in a total of 151,200 frames. Zoarcid fishes (eelpouts), red crabs (*Paralomis multispina*) and finally, snails (*Buccinum soyomaruae*), were recognized as the most recurrent species within the Region of Interest (ROI).

### 3.1. Error Estimation of Automated Video-image Analysis

On the training set of 10.000 images, the trained operator identified 2,563 frames (25.6%) with moving organisms within the ROI. The object identification by KNN0 without thresholding (i.e., without EDT) correctly identified moving animals in 2,087 images (81.4% of frames with organisms). Differently, moving animals in 18.6% of frames were not identified and extracted as directly checked by the trained operator. Also, 7.0% of frames with no animals within the ROI were considered as having moving organisms (i.e., false positive). More than 1,000 images (3.0%) were eliminated automatically due to excessive background noise.

The object classification after KNN thresholding produced better results of automated motion detection. The error in the single species classification and null objects (false objects) was reported in Table 2. Although the number of images with moving animals not recognized by the tracking analysis (Type-1 error) increased, the thresholding drastically reduced the cases of false positive identification (Type-2 error). Only the 0.4% of frames without organisms was considered as having moving animals in their ROI. The 53.5% of frames with moving animals were identified as not having it. Also, fishes, crabs, and snails were never misclassified each other. Fishes presented the highest level of correct classification (77.92%). The 42.96% of snails were not classified as null. Only 0.9% of null objects were classified as real objects (0.01% for fishes and 0.89% for snails).

**Figure 4.**
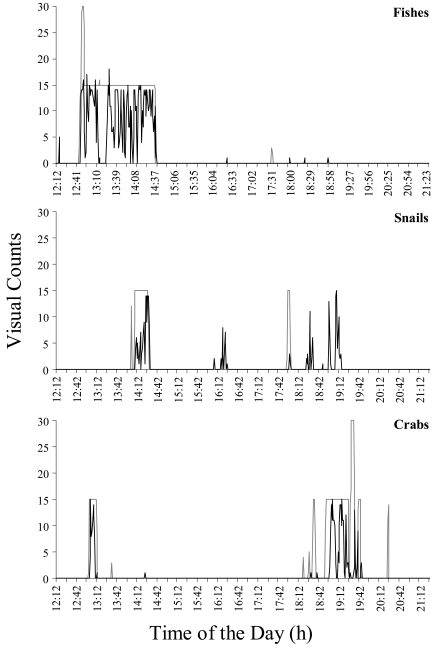
Time series of visual counts per minute as estimated by automated video-image analysis (black line) and by manual classification from the trained operator (grey line) are presented in order to account for the reliability of applied methodology.

We obtained time series of data with no missing values, and with no variations in sampling frequency. Figure 4 reports the comparison of time series in visual counts for eelpout fishes, snails, and finally crabs during the first 11^th^ hours of video recording, as obtained by direct operator counting and automated video-image analysis. Time series are almost similar as a proof of the validity of the method. On a total of 9,990 frames (i.e., a frame per 4 s on 11.1 h), automated video-image analysis recognized moving eelpouts in 105 frames, while the trained operator identified animals in 118 frames. We computed an efficiency index (in %) to evaluate robustness of our method. The matching between visual and automatic counts for each species per minute is equal to: 96.4% for eelpouts, 91.9% for snails, and 95.8% for crabs.

### 3.2. Rhythmic Behaviour Analysis

**Figure 5.**
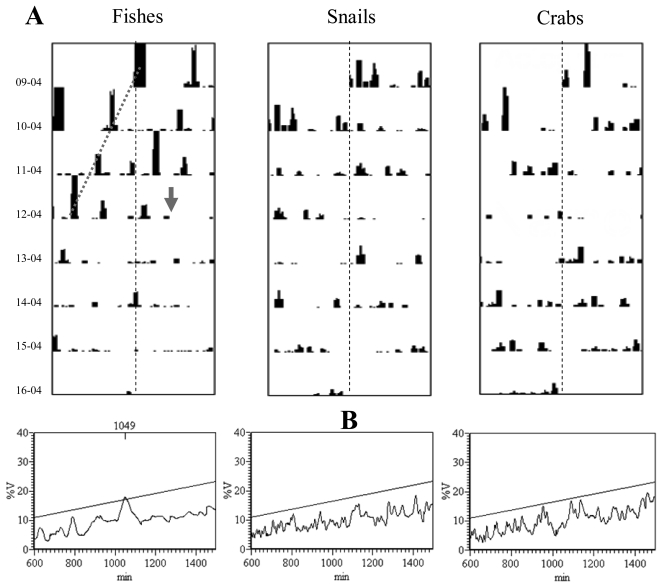
Double-plot actograms (A; vertical dashed line is the 24-h based limit) depicting time series of visual counts for eelpout fishes, red crabs, and snails, within the video camera region of interest. Observations show rhythmic fluctuations only for fishes, being the other two invertebrate species of the cold-seepage community arrhythmic. This is confirmed by periodogram analysis (B) identifying a significant periodicity only for eelpout.

Double-plot actograms referring to the number of observed moving eelpouts, crabs, and snails are presented in Figure 5. Complex rhythmic patterns appeared with different strengths in the corresponding time series, being apparently marked in fishes (Figure 5A). As revealed by the program analysis (Figure 5B), eelpouts rhythmic behaviour presented a periodicity of 1,049 min (equal to 17.5 h). Crabs and snails did not present any discernible rhythmicity in their temporal plots.

**Figure 6.**
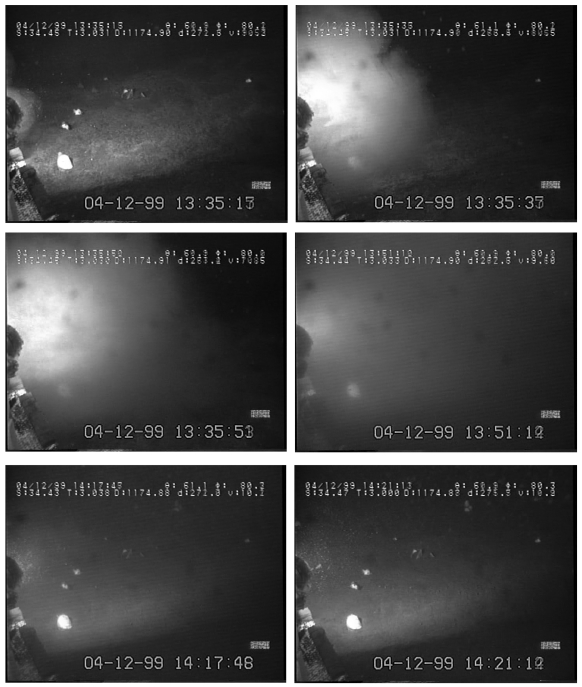
Consecutive frames portraying the development of a mudflow. Water turbidity increased within few minutes after the mudflow front arrivals and a high turbidity condition, obscuring video recording, persisted over approximately one hour. After that event, a disruption in reported rhythms in eelpout behaviour (as indicated by time series of visual counts) occurred.

During the fourth day of video recording, a sweeping mudflow obscured video recordings from approximately 13:35 to 14:21, hence lasting close to one hour (Figure 6). Water turbidity acquired its maximum within few minutes form the arrival of the mud front (visible in the camera field as a black shadow). Time series of visual counts for the three species showed an amplitude reduction from that moment on (the day: 12-04; see Figure 5A).

### 3.3. Displacement Trajectories Analysis and Other Ethological Observations

**Figure 7.**
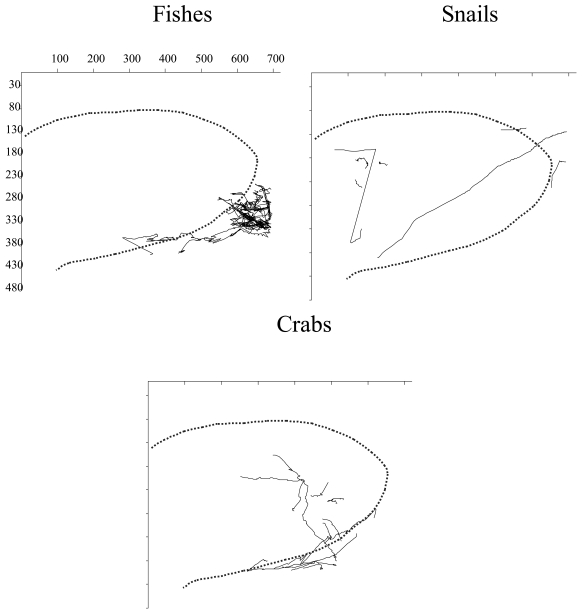
Different trajectories for eelpout fishes, red crabs and snails as detected within the region of interest. The dashed enclosure indicates the bottom area illuminated by video camera lamps.

The tracking analysis evidenced the occurrence of interspecific differences in the displacement behaviour of detected species in relation to the *xy*-ROI coordinates and hence the light field of video-camera (Figure 7). Eelpout fishes were present at the border of the illuminated area while crabs and snails showed a less influenced response. Snails chiefly assumed rectilinear trajectories within the video camera light field as well as snails. While crabs presented a zigzag patters of displacement, snails displaced in a more rectilinear fashion.

## 4. Discussion

In this study, we have presented a novel protocol of automated video-image analysis for the identification of species and the study of their behavioural rhythms. We could successfully identify and then track the movement of most abundant elements within the cold deep local community of Sagami Bay: Zoarcid fishes (eelpouts), red crabs (*Paralomis multispina*), and snails (*Buccinum soyomaruae*). We presented data suggesting a potential response of community elements to flow changes at inertial periodicity. We also quantified interspecific differences in animal behaviour in relation to the video camera light field and unpredictable contingent phenomena such as mudflow events.

With rapid improvements in underwater video-technology, automation is becoming an important bottleneck for analysing data [25]. The use of multivariate morphometry in automated video-image analysis targeting species recognition was not often performed. We implemented our automated analysis protocol also by an additional step of comparison of automated results with those provided by a trained operator [26]. In order to do so, a reference library on observed shapes was created. That procedure successfully incremented the efficiency of our technique suggesting that expert supervision associated to baseline automation can be an essential step to increase species recognition performances. This proposes a future scenario for marine biology research where the increment in classification performances of remote video devices is constantly accompanied by the constant tuning (by trained operators) of those class categories used for object classification.

In this study, the analysis of trajectories indicated a different status in the activity of eelpout fishes, crabs, and snails within the light field (Figure 7). Deep-sea observations usually employ bright lights that can damage the optic apparatuses of local fauna for the action of emitted photons on animals' too sensitive visual systems (reviewed in [27]). Light avoidance of demersal deep-water and deep-sea species is then a common phenomenon [9]. Eelpout fishes avoided the light field but our recording methodology did not impair the detection of clearer swimming rhythms in relation to the other two targeted species. Red crabs and snails showed trajectory fully encompassing the light field as an indication that, differently from fishes, these invertebrates are less affected by direct illumination exposure. These results are similar to those reported for fishes by [9], where animals' counts increased within the ROI at moments of light off (and infrared on). Unfortunately, infrared technology was not installed on the video-camera station of Sagami at its deployment in 1993 [28].

The physiological and behavioural response of deep-sea fishes and crustaceans to rhythmic changes in bottom currents was already characterized in the Atlantic [5,29-31]. Those studies employed different sampling systems but behavioural data were not obtained from automated video-image analysis. Also, time series of visual observations were not treated with time series analysis protocols, as typical of chronobiological studies. We emended that issue and we reported a weak behavioural response of fishes to flow changes at inertial frequency, fitting observational oceanographic data at the latitude of Sagami Bay [32].

Our study focused on the rhythmic behaviour of demersal species with different typologies of displacement, a fact of importance for the ecological evaluation of behavioural results. For fishes, behavioural observations suggest an alternate pattern of swimming and lying over the bottom in association to water speed increases and decreases, which affect the rate of visual counting.

The study of behavioural rhythms with underwater permanent stations can be criticized when aimed to the description of species activity rhythms based on time series of visual observations. The recorded visual count patterns may be globally arrhythmic for the random phase agreement in the activity rhythms of different individuals within the local population. Anyway, dominant geophysical cycles modulate the rhythmic behaviour of several individuals in a similar fashion and synchronism should be than expected at populational level [33]. Unfortunately, with our protocol, we could not distinguish different individuals within each species category (eliminating hence the recounting of the same individual). We had also the added difficulty of image processing from digitized old VH-S tapes. The behavioural rhythm regulation in deep-sea areas will be more feasibly studied by video-filming with high density cameras of confined (i.e., within cage) fishes or sessil organisms such as giant tube worms (*Pogonophora* and *Vestimentifera*).

## 5. Conclusions

Our data indicate that video technology can be successfully applied to deep-sea studies on species identification and behavioural rhythms characterization. Neuroethological research is currently based on the assumption that with a better process of behavioural tracking, a deeper understanding of neuronal controlling processes can be acquired [34]. In this sense, video-image analysis is already replacing telemetry and actography in laboratory studies since it is less expensive, more compact and hence, more technologically feasible from the point of view of hardware complexity [16]. The same consideration could be applied for field studies. The analytic protocol presented in our work can be potentially used with several types of video sources form very different environments, where permanent stations are acquiring (or may acquire in the next future) footages of very long duration (e.g., months).

## Figures and Tables

**Table 1.** Logical conditions for the class attribution with the KNN0 classification.

**RGB class**	**FD class**	**RGB minimum Euclidean Distance**	**FD minimum Euclidean Distance**	**Final class attribution**
Eelpouts	Eelpouts	< 4.5	< 0.2	Eelpouts
Crabs	any	< 4.5	any	Crabs
Snails	Snails	< 4.5	< 0.2	Snails

**Table 2.** Absolute values and percentage of correct classification by tracking analysis of displacing organisms for each selected species is reported with the percentage of species attribution to the Null category (false object). Type-1 and Type-2 errors are also reported.

	**Eelpouts**	**Snails**	**Crabs**	**NULL**	**Type-1**	**Type-2**
Eelpouts	1320 (77.92%)	0 (0.00%)	0 (0.00%)	374 (22.08%)	22.08%	0.01%
Snails	0 (0.00%)	174 (42.96%)	0 (0.00%)	231 (57.04%)	57.04%	0.89%
Crabs	0 (0.00%)	0 (0.00%)	610 (63.02%)	358 (36.98%)	36.98%	0.00%
Null	1 (0.01%)	66 (0.89%)	0 (0.00%)	7370 (99.10%)		
